# Methods to Determine the Lagrangian Shear Experienced by Platelets during Thrombus Growth

**DOI:** 10.1371/journal.pone.0144860

**Published:** 2015-12-14

**Authors:** Isaac P. Pinar, Jane F. Arthur, Robert K. Andrews, Elizabeth E. Gardiner, Kris Ryan, Josie Carberry

**Affiliations:** 1 Department of Mechanical and Aerospace Engineering, Monash University, Melbourne, Victoria, Australia; 2 Division of Biological Engineering, Faculty of Engineering, Monash University, Melbourne, Victoria, Australia; 3 Australian Centre for Blood Diseases, Monash University, Melbourne, Victoria, Australia; Queen Mary University of London, Blizard Institute, UNITED KINGDOM

## Abstract

Platelets can become activated in response to changes in flow-induced shear; however, the underlying molecular mechanisms are not clearly understood. Here we present new techniques for experimentally measuring the flow-induced shear rate experienced by platelets prior to adhering to a thrombus. We examined the dynamics of blood flow around experimentally grown thrombus geometries using a novel combination of experimental (*ex vivo*) and numerical (*in silico*) methodologies. Using a microcapillary system, platelet aggregate formation was analysed at elevated shear rates in the presence of coagulation inhibitors, where thrombus formation is predominantly platelet-dependent. These approaches permit the resolution and quantification of thrombus parameters at the scale of individual platelets (2 μm) in order to quantify real time thrombus development. Using our new techniques we can correlate the shear rate experienced by platelets with the extent of platelet adhesion and aggregation. The techniques presented offer the unique capacity to determine the flow properties for a temporally evolving thrombus field in real time.

## Introduction

Platelets are small anucleate blood cells that are crucial in preventing major blood loss due to injury, with the capacity to rapidly adhere and aggregate on the blood vessel wall in flowing blood (thrombus formation). The variable forces that platelets are exposed to in the bloodstream critically impact on platelet function. Significant improvements towards anti-platelet therapies have been hindered by incomplete knowledge of the underlying biological and biomechanical mechanisms driving platelet adhesion and aggregation into consolidated thrombi. For a long time the focus in this field has been on blocking the ligand-receptor interactions or activation pathways that mediate platelet aggregation and thrombus formation [1]. Consequently, anti-platelet therapeutics increase bleeding risk. Understanding how platelet activation is modulated by exposure to elevated shear rate is imperative for the design of new reagents that potentially curb, rather than block, platelet function pathways. Given that cardiovascular diseases involve thrombus formation in regions of high and disturbed shear rate, reagents that target platelet function under these conditions are required.

Many groups have studied thrombus formation under shear conditions [2–7]. Nesbitt *et al*. showed that platelets respond rapidly to changes in local shear stresses within a blood vessel [2]. In the absence of other biochemical interactions, subtle variations in shear stress can promote platelet adhesion and thrombus formation. Additionally the environment surrounding a growing thrombus has been shown to promote the formation of further platelet aggregates [8]. Currently very little is known about how temporal changes in shear rate experienced by the flowing platelet affect platelet adhesion, aggregation and thrombus stability (embolism). In order to address these questions, our aim was to use a combined approach to simultaneously measure blood flow properties and growth. Determining Lagrangian flow properties (observing what happens to the platelet in the reference frame of the platelet) without the use of numerical flow experiments, i.e. using physical experiments, is extremely difficult, if not impossible. Extracting Lagrangian flow properties (i.e. considering the motion of individual platelets as they move through the microchannel) experienced by platelets as they move in the blood flow allows for parameters such as shear rate, velocity and changes in velocity to be determined for individual platelets. It is then possible to determine how these platelets respond to the flow properties by measuring the rate at which they adhere. Separate measurement of the flow field and thrombus growth properties does not allow for correlation between what platelets experience in the flow and their subsequent response within the one *ex vivo* experiment, therefore a novel combined approach is required. Such an approach would be suitable for other applications (e.g. time variable cell growth in cultures) to quantitatively evaluate changes occurring over time in a three-dimensional (3D) environment.

A number of currently available techniques can measure or control the shear rate experienced by platelets [9–10]. Previous techniques for measuring the shear rate platelets undergo during thrombus formation have focused on micro-particle image velocimetry (μPIV) or direct numerical simulation of the flow [10–11]. Blood flow is a challenging environment for μPIV and consequently the obtainable flow fields are either 2D or 3D time averaged without providing real time thrombus growth data. μPIV provides reasonably accurate shear rate values however is highly dependent on resolution. Additionally, the interaction between PIV seed particles and red blood cells can alter the velocity profile [11]. Conversely, numerical simulation provides highly accurate shear rate calculation, with the time taken to calculate the shear field varying significantly with the number of thrombi present [12].

In the present study, we examine the dynamics of blood flow around experimentally grown thrombus geometries using a novel combination of experimental (*ex vivo*) and numerical (*in silico*) methodologies [2–3]. We use a microcapillary system, in the absence of coagulation, to analyse human platelet aggregate formation at elevated shear rates, and develop a new method to extract instantaneous time-corrected reconstructions of a growing geometry using confocal imaging. The study focuses on platelet interactions with a temporally evolving thrombus. Effects of adhesion or aggregation proteins and red blood cells (RBCs) are not considered. As whole blood is used, the RBCs travel predominantly along the centre of the microcapillaries (~200 μm height) while the peak height of the thrombi are much lower (~30 μm height). The presence of RBCs in the flow tends to push platelets towards the microcapillary walls (where thrombus growth is being analysed), thereby increasing the platelet concentration at the wall. Additionally, the Fahraeus- Lindqvist effect should not be of great significance as the hydraulic diameter of the micocapillaries is relatively large (0.36 mm) for this effect to have a great implication. As such there should be no effects on the viscosity of the blood. Together, these studies reveal how the shear rate profile experienced by potentially adherent platelets affect thrombus growth, with implications for potential regulation of thrombosis associated with pathological shear flows.

## Methodology

**Fig 1:**
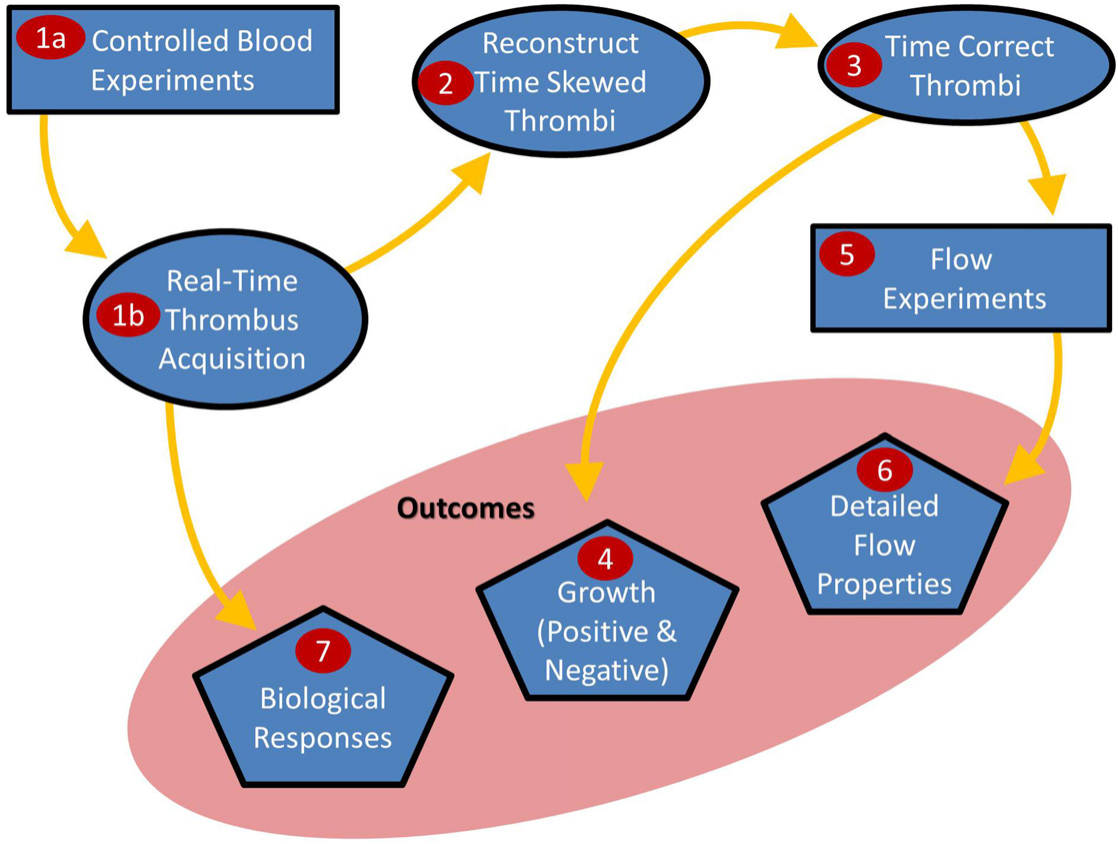
Summary of the combined approach process. Controlled blood flow experiments are performed to form thrombi in real-time (i). These thrombi are reconstructed into a 3D surface (ii) and time-corrected (iii) to account for the duration it takes to image a thrombus. Time-correcting the thrombi allows for accurate growth rates to be calculated and spatially mapped (iv). Flow experiments (v) are completed to determine the detailed flow properties (vi).

Here we present new techniques to correlate the Lagrangian flow properties (particularly shear rate) experienced by platelets in blood flow with subsequent thrombus growth. This is a multiple stage process utilising both existing and new techniques as depicted in Fig 1.

### 1.Controlled Blood Experiments

A 100-mm long, rectangular, glass microcapillary with cross section 2 x 0.2 mm (width x height) was coated with bovine type 1 collagen (100 μg/mL) then rinsed with Tyrode’s buffer (138 mM NaCl, 5.5 mM glucose, 12 mM NaHCO_3_, 2.6 mM KCl, 0.36 mM NaH_2_PO_4_, 1.8 mM CaCl_2_, 0.49 mM MgCl_2_.6H_2_O, pH 7.4), following the experimental procedure of Tolouei *et*. *al*. [3]. The microcapillary and associated tubing (ID 0.8 mm) were perfused with Tyrode’s buffer to prevent air bubbles. Whole blood from healthy human donors was collected after written consent, in accordance with the Declaration of Helsinki, and the study was approved by the Monash University Standing Committee for Research in Humans. Blood from two donors without any prior medication use, was used over four experiments. Blood was combined with (final concentration) 800 U/ml hirudin anticoagulant and platelet membranes stained with a fluorescent dye (DiOC_6_), to allow platelets to be imaged by confocal fluorescence microscopy (Nikon A1 Confocal Fluorescent Microscope, Apochromat 40x Water Immersion lens, 512 x 512 pixel image resolution acquired at 30 fps) as previously performed by Yuan *et al*. [13]. Blood was pulled through the microcapillary using a syringe pump (Harvard Apparatus PHD2000) at a mean flow rate of 1.44 ml.min^-1^, generating a wall shear rate of 1800 s^-1^. This shear rate corresponds to nonphysiological arterial shear rates measured in coronary vessels.

Whole blood was transfused through the system for 3 minutes, allowing for the growth of thrombus geometries within the microcapillary. Images were acquired using 0.5 μm thick slices (pixel density 0.31 μm/pixel) with no time delay between image stacks and two averages per slice. For projections such as platelet pseudopods to be observed, a slice thickness of 0.1 μm would be required, resulting in significantly longer scan times, therefore reducing overall temporal resolution. Spherical aberration of the lens can result in the lower slices needing post processing to determine the true 0 z-position slice. In order to ensure the entire thrombus was captured, scanning began below the interface between the bottom of the thrombus and the bottom of the microcapillary. The image acquisition was optimised to maximise temporal resolution while maintaining sufficient image quality for thrombus reconstruction. It is also possible to detail internal components (i.e. individual platelet boundaries) of a single thrombus however this study focuses on the overall thrombus surface across a field of thrombi.

### 2.Reconstruction of Time-Skewed Thrombi

The images captured by the confocal microscope were converted from the native 12-bit colour spectrum to 8-bit images in order to apply a greyscale pixel based intensity threshold across each slice. The edges of each individual thrombus were isolated using in-house segmentation software and saved as separate images. The boundaries around each thrombus were stacked vertically to form a 3D point cloud of the thrombus. A 3D surface was generated to enclose these points using Avizo software [14] utilizing highly optimised surface reconstruction algorithms combined with variable extent surface smoothing to allow sufficient control over the generation of complex surface models.

Utilising Avizo to completely reconstruct the confocal data produced “ripple” artefacts between z-slices which did not accurately represent the thrombus surface. In-house software was developed to filter these ripples and the surfaces were validated using a mathematically-derived trapezoid [S1 Text].

During the thrombus growth phase, image acquisition speed is critical and taking either more slice averages or increasing the vertical slice resolution adversely increased the time it took to acquire a set of images. The slice thickness used in the confocal imaging was optimised by considering both the size of structures being measured (platelet ~ 2 μm) and the requirement to minimise scan time to resolve temporal variations; consequently a slice thickness of 0.5 μm was used.

### 3.Time-Correction of Thrombi

**Fig 2:**
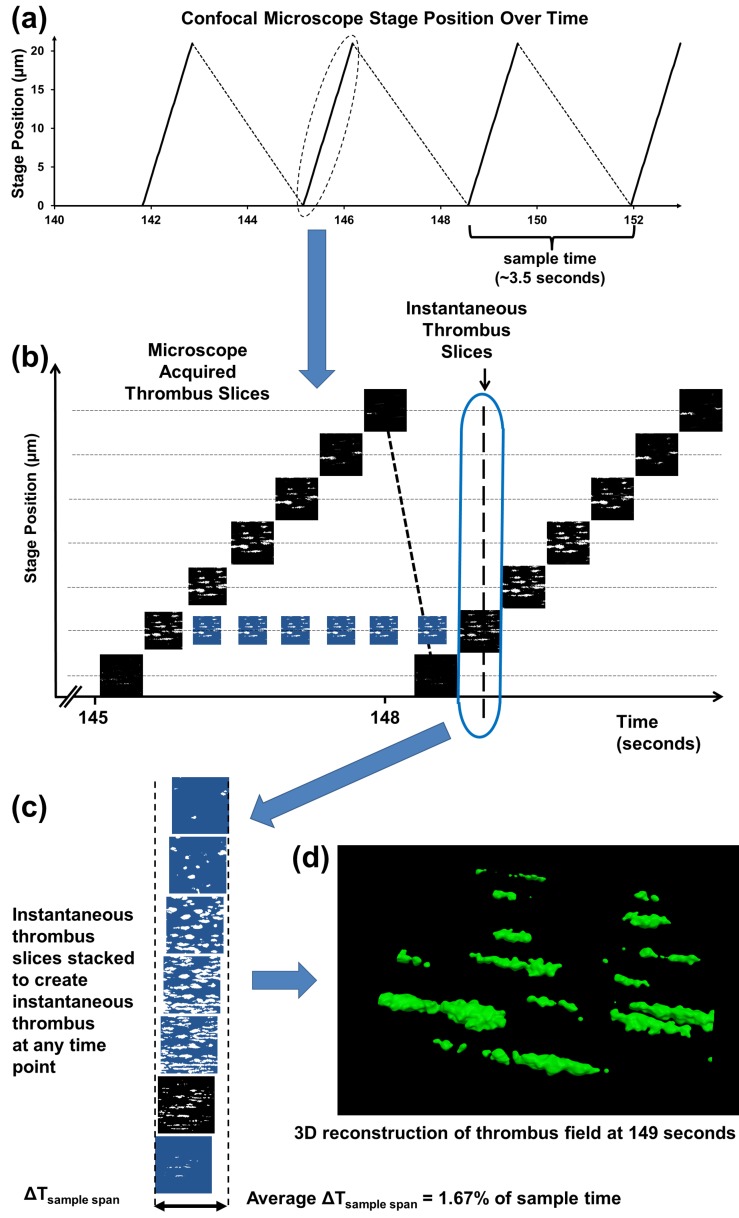
Time-corrected thrombus reconstruction process. **(**a) Image metadata showing microscope stage position over time. (b) Individual slices have original acquisition times assigned from metadata. Acquired confocal images are in black; morphed intermediate images are in blue (c). Slices corresponding to the 149-second timepoint are extracted and stacked vertically forming an instantaneous z-stack which is reconstructed (d) to produce the thrombus field at 149 seconds.

Using our optimised settings, it took 3.5 seconds to vertically scan through a typically sized thrombus [75 μm (L) x 12μm (W) x 30 μm (H)], as shown in Fig 2A. It took 1.2 seconds to scan from the 0 z-position to the top of the stack and 2.3 seconds to transfer data to the computer. Over the 3.5 second z-stack acquisition, the thrombus is continually growing which impacts on the accuracy of the measured thrombus geometry. A thrombus reconstructed directly from a single z-stack represents a skewed average of the instantaneous thrombi present while the z-stack was being acquired. For example, if a growing thrombus was scanned from the bottom upwards the acquired lower portion will miss much of the volume from growth occurring during the scan, while the upper portion will capture most or all of the extra growth volume. In order to accurately study the rates at which platelets adhere to thrombus geometries, the growth occurring during the z-stack acquisition should be corrected.

A new algorithm based on morphing techniques was developed to account for the significant growth occurring during the time it took to acquire the z-stack. The algorithm requires time resolved knowledge of the microscope stage position, Fig 2A, commonly provided in the metadata file. We morphologically transformed the 2D images acquired in successive scans at each z-level, allowing resampling of the thrombus geometry at a single timepoint. A modified active contour, level set method [15] was used to morphologically generate the intermediate confocal slices at each z-level as shown in Fig 2B. Using this technique allows for a “snapshot” of the thrombus at single timepoints from which all the relevant growth properties can be accurately calculated.

The active contour method is an energy minimization technique that aims to determine the outline of an object from a 2D image which in most cases tends to have significant noise levels. Normally this method is used in image segmentation with a basic initial shape such as a circle or square that is minimized to form a contour around a particular object. The implementation begins with the outline of a thrombus at timepoint 1 as the initial contour, which is then deformed in various directions towards the outline of the thrombus at timepoint 2. The subsequent contour evolutions form the intermediate thrombus boundaries. Only when the energy associated with the contour is completely minimized will all the points along the contour represent the thrombus at timepoint 2 [S1 Text].

The images acquired from the confocal microscope are sorted into time ordered image stacks based on the time stamps from the microscope’s metadata file (Fig 2A). The algorithm starts at the bottom slices between consecutive time stacks and selects the segmented thrombus edges from the first stack (timepoint 1) as the initial condition and iteratively minimizes the contour energy until it arrives at the contour from the second stack (timepoint 2). The intermediate frames are stored and assigned a real time value (Fig 2B & 2C). The process is repeated until the top slices are reached, at which point the algorithm starts again from the bottom slices of the next stack pair. The timepoints before the end of acquisition of the first stack and the start of acquisition of the last stack are not able to be utilised since there are no slices with which to correct time. Instantaneous snapshots of the thrombus geometries are available at all other timepoints by sampling contour slices vertically at the timepoint at which an instantaneous thrombus geometry is required to form an instantaneous z-stack. As the intermediate frames are sampled at defined increments their time stamp may not match exactly the instantaneous sample time and the image from the nearest timepoint must be used. Thus the instantaneous z-stack contains images from a very small time band ΔT_sample span_. For our experiments ΔT_sample span_ was 56.3 milliseconds or 1.67% of the time taken to acquire the z-stack. Finally the instantaneous z-stack is reconstructed into a 3D surface (Fig 2D). This technique can be applied to individual thrombi or a thrombus field.

#### Validation of Time-Correction Technique

**Fig 3:**
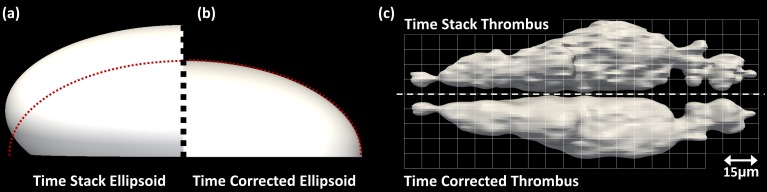
Comparison between real-time surfaces and time skewed surfaces. Comparison between ellipsoid reconstructions constructed using (a) real-time corrected ellipse slices and (b) ellipse slices derived from time skewed slices. (c) Comparison between time stack thrombus reconstruction (top) and real-time thrombus reconstruction (bottom) mirrored through the central plane.

Validation of the time-correction technique was performed using a mathematically constructed semi-ellipsoid surface grown in time at different rates along each axis. The resulting surface grew in a non-linear manner to resemble thrombus growth. The growing ellipsoid was sampled by taking sequential z-slices, moving upwards, as the ellipsoid grows, i.e. sampling the growing surface in the same way the z-stack of a growing thrombus is acquired by confocal imaging. The volume of the ellipse grew by 12% during the time the z-stack was acquired, mimicking thrombus growth. The ellipsoid surface was reconstructed in two ways: first, without correction directly from the z-stack and second, using the time correction technique to acquire an instantaneous time-corrected surface at a timepoint at the centre of the z-stack time. The results are shown in Fig 3A & 3B) where the left half surface is without correction, the right half surface is the time-corrected instantaneous surface and the dashed red line is the mathematically constructed surface at the same timepoint as the instantaneous surface. As expected, the uncorrected surface is smaller at the bottom and larger at the top demonstrating the growth that occurs during the z-stack acquisition, while the time-corrected surface corresponds well with the mathematical surface.

The technique was also applied to real-time thrombus data to compare a time stack reconstruction with a time-corrected instantaneous thrombus (at the centre of the z-stack time). Fig 3C shows the mirror image comparison of the resulting surfaces. The time stack reconstruction is both narrower (2%) and taller (3%) than the time-corrected thrombus and shows larger growth at the top of the thrombus surface which is not present in the instantaneous real time thrombus surface. These regions of growth have occurred after the point in time that the real time thrombus is representing and therefore should not be evident in the corrected reconstruction.

The algorithm we have developed to correct for growth during z-stack acquisition can be applied to a variety of biological systems where the subject is growing or moving over the duration of a scan.

**Fig 4:**
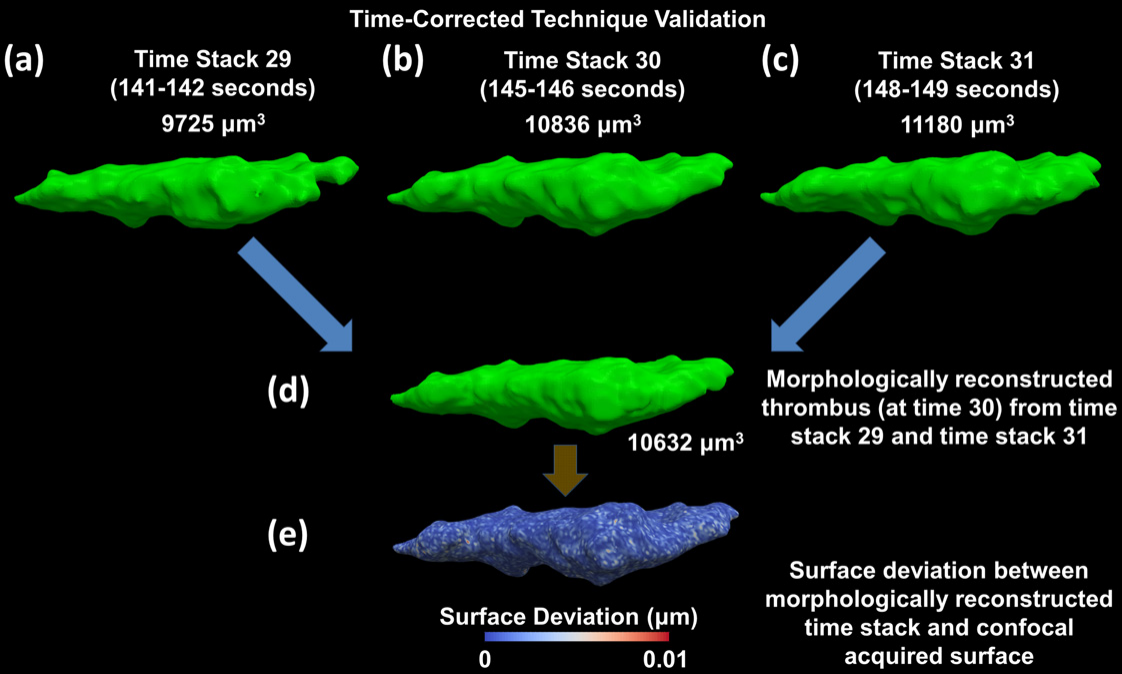
Validation of time-correction technique using two time stacks to reconstruct the intermediate time stack. (a), (b), (c) Time stacks 29, 30 and 31 are all acquired using confocal microscopy and reconstructed. (d) Time stack 29 and 31 are then used to reconstruct the thrombus surface at the intermediate time stack 30. (e) The deviation between time stack 30 acquired using confocal microscopy and the morphologically reconstructed time stack 30 measured as a spatial distance (Maximum spatial deviation < 10^−2^ μm). Blue indicates a deviation of 0 μm, while red indicates a deviation of 10^−2^ μm.

The morphing algorithm which is central to the technique to time-correct a growing thrombus is further validated by using two known surfaces to produce an intermediate known surface. Fig 4(A), 4(B) and 4(C) show a thrombus at three different points in time which have been acquired using confocal microscopy, as described above. Using the surfaces from an initial and final time stack, a surface at the timepoint corresponding to the intermediate time stack is morphologically reconstructed (Fig 4D). The surface from the intermediate time stack (Fig 4B) and the surface morphologically generated at the same timepoint (Fig 4D) are compared based on an overall volumetric deviation, which was 1.9% between the two surfaces. Additionally, a spatial deviation was calculated between the two surfaces which resulted in a maximum spatial deviation of 0.01 μm (Fig 4E). In an ideal case there should be no deviation between these surfaces however due to the large time difference between initial and final time stacks (greater than 8 seconds) this deviation is well below the confocal image pixel resolution and is considered to be negligible.

### 4.Thrombus Growth

**Fig 5:**
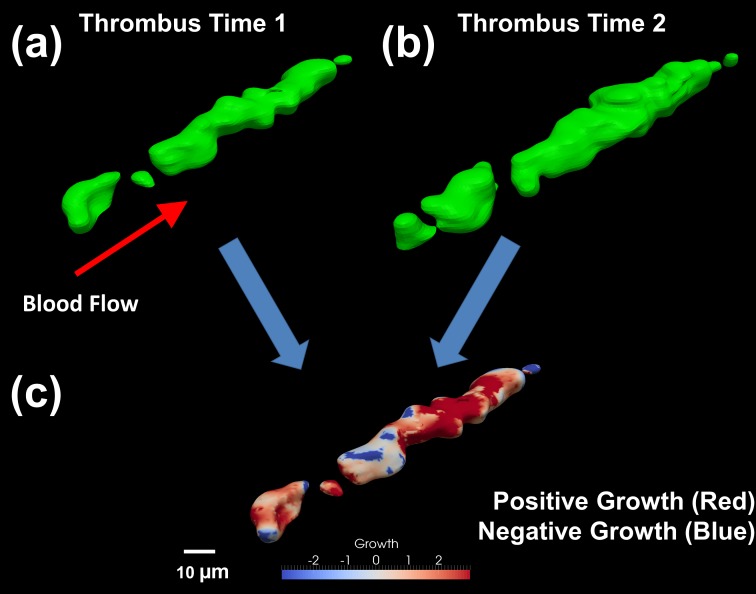
Determining regions of high growth or consolidation and quantifying the amount of growth between two points in time. (a) The reconstructed thrombus surface at time 1 (100 seconds into growth phase). (b) The reconstructed thrombus surface at time 2 (135 seconds into growth phase). (c) Developed algorithm indicates which regions have grown (shown in red) and those regions which have consolidated or retracted (shown in blue). Normal vectors are calculated between the surface at time 1 towards the surface at time 2 from which a Euclidean distance is calculated to give the magnitude a particular point on the surface has either grown or consolidated between the two points in time. The normal vector magnitudes are mapped onto the surface at time 1 highlighting regions of high growth or consolidation.

Since platelet aggregate (thrombus) formation can be subject to simultaneous platelet adhesion and loss in addition to thrombus retraction, both positive and negative growth must be considered. These measurements are crucial in determining the levels of thrombosis and embolism. Aggregations are being considered in the form of platelets attaching to other platelets on the thrombus surface or platelets attaching to coagulation factors present on the thrombus surface. Thrombus growth is determined by calculating the Euclidean distance (straight line distance in 3D space between 2 points) between two thrombus surfaces (Fig 5A & 5B). Growth in this context includes volume changes due to other factors such as RBCs in addition to free flowing platelets. The technique presented calculates positive or negative growth from the 3D thrombus surface by projecting surface normals both inwards and outwards from the thrombus surface at time 1 to intersect with the surface at time 2. Each surface point has two opposing normal rays projected to ensure both positive and negative thrombus growth is captured. The corresponding magnitude and direction of growth between the surfaces are contour mapped onto the original thrombus surface (Fig 5C). Red contours indicate areas of high positive growth (adhesion) while blue regions represent losses in the thrombus surface (embolism or consolidation). Quantifying thrombus growth data allows for direct comparisons to be made between growth and the shear properties experienced at precise locations on the thrombus surface.

### 5.Flow Experiments

New techniques were required to quantify the flow properties (shear rate/velocity) that an incoming platelet experiences before interacting with the thrombus surface. The steady flow of blood around a thrombus in a 100mm (L) microcapillary (cross section 2 mm x 0.2 mm), was modelled using the OpenFOAM package [16]. Blood is considered as an incompressible and Newtonian fluid and a viscosity of 2.8cP is used in the numerical analysis. In order to replicate the *ex vivo* flow conditions, the numerical model matched the Reynolds number (Re ~7.3 based on the microcapillary hydraulic diameter) and aspect ratio (AR = capillary width/capillary height = 0.1) [3]. A solution to the incompressible Navier-Stokes equations to steady state was obtained through the OpenFOAM CFD solver package (pressure and velocity residuals less than 10^−10^) [S1 Text]. The SIMPLE algorithm was used to determine both the velocity and pressure fields of blood flowing through the capillary at a flow rate of 1.44 ml.min^-1^, corresponding to a wall shear rate (γ_w_) of 1800 s^-1^. The local shear rate was calculated as follows:

$$\gamma_{local}=\sqrt{2∙(D:D)}$$ (1)

where D is the strain rate tensor and U the velocity vector field defined by,

$${D=\nabla U+(\nabla U)}^{T}$$ (2)

The upstream region of the microcapillary was determined to be sufficiently long enough to obtain a fully developed steady state Poiseuille flow by allowing transients in the flow field to damp out to below 2%, while the outlet was set to a constant pressure gradient. The top, bottom and sides of the microcapillary and thrombus surface had a zero velocity Dirichlet boundary condition imposed. A polyhedral finite volume mesh was utilised throughout the domain with a refinement region 200 μm radially around the thrombus geometry. The overall domain mesh consisted of approximately 17 million elements, with the thrombus surface accounting for around 15.8 million elements.

### 6.Detailed Flow Properties

The shear forces that individual platelets experience are calculated directly from the numerically generated flow field. It is assumed that prior to adhesion the platelet follows the flow pathlines. A thrombus interaction region (TIR), defined as 10% of the thrombus length upstream in the flow direction, is used to represent the region where platelets experience shear rate gradients from interactions with the thrombus surface. Calculation of the flow-induced forces experienced by platelets before interaction with a particular location on the thrombus surface is accomplished by taking points 1μm above (along the normal vector) the reconstructed thrombus surface to construct pathlines backwards in time. Platelets are currently not modelled within the numerical simulations as particles. Pathline trajectories are calculated by integrating the velocity vector field towards the inlet of the numerical domain using a fourth order adaptive Runge-Kutta method [17]. A typical thrombus contains approximately 40,000 surface points. Spatial paths taken by individual platelets and the corresponding shear rates were determined as a function of time with the maximum spatial error along pathlines maintained below 0.001 μm. At 1 μm above the thrombus surface (based on the nominal platelet diameter of ~2 μm), a platelet is deemed to be in physical contact with the thrombus. In order to avoid the Dirichlet boundary condition is imposed on the thrombus surface (U_surface_ = 0 ms^-1^), the pathlines originate from points 1 μm above the thrombus surface. Properties, such as shear rate at adhesion (1 μm above the surface), peak shear rate along the pathline and average shear rate along the pathline, which describe the hemodynamic forces a platelet experiences at and prior to adhesion, are projected onto the thrombus surface and represented as a contoured surface. The forces being mapped onto the thrombus surface resemble the forces a platelet 1 μm above the thrombus surface would experience in that particular location. These hemodynamic properties can then be correlated with the local growth of the thrombus surface.

## Results and Discussion

The fluid mechanical mechanisms which govern platelet adhesion in various hemodynamic environments can be better understood through the combination of the techniques presented. Most notably we can measure how Lagrangian flow properties (including shear rate experienced by a platelet in the blood flow prior to adhesion) contribute to the subsequent growth of a thrombus. While the methods shown have been illustrated using a single thrombus geometry, thrombus growth and the shear rate experienced by platelets approaching an entire field of thrombi can also be determined. Additionally, the techniques demonstrated can also be applied to pulsatile blood flow where temporal resolution is adequate for oscillating flows up to 3 Hz although with a reduced pixel density of 0.43 μm/pixel.

**Fig 6:**
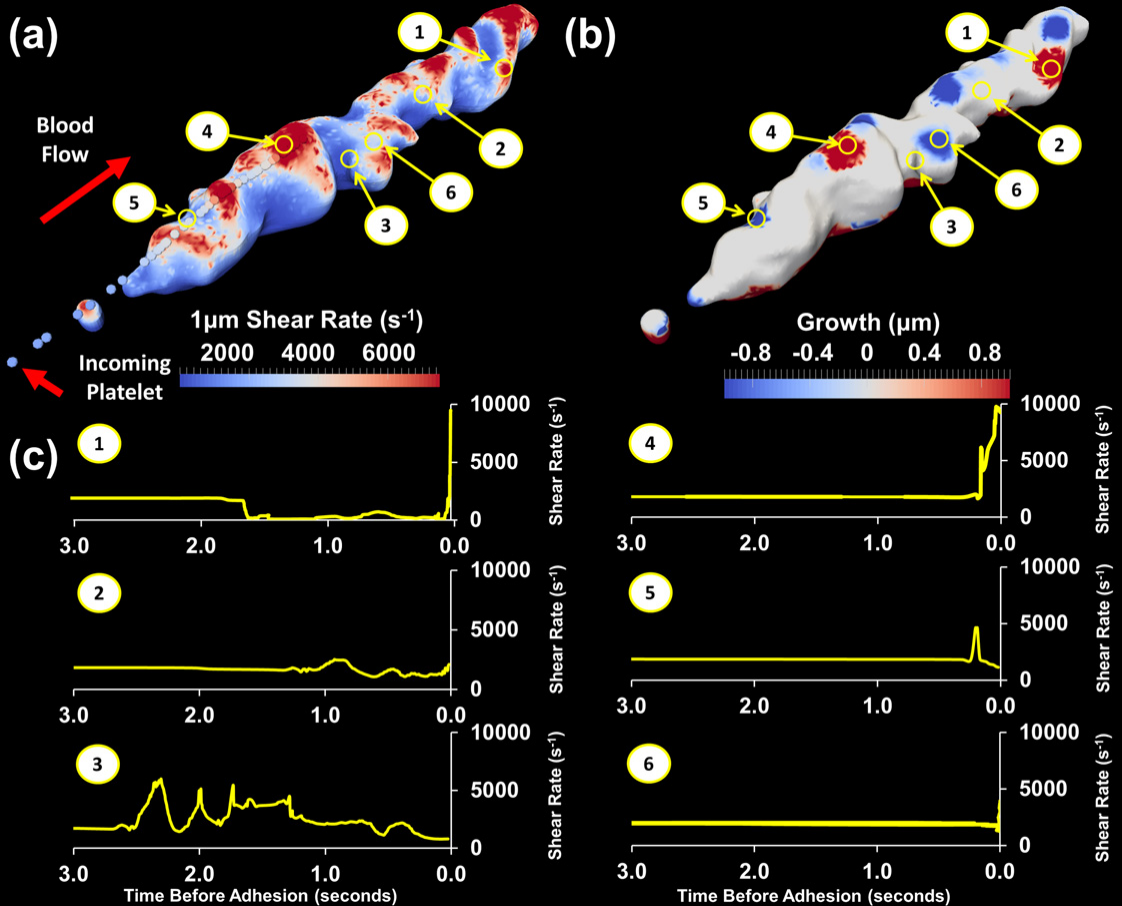
Thrombus growth and shear rate mapped onto original thrombus surface with shear rate experienced by individual platelets. **(**a) Shear rate 1 μm above surface mapped onto original thrombus surface. Spheres represent the path a platelet landing in a high shear rate region follows, coloured by shear rate experienced (b) Original thrombus surface with regions of growth (shown in red) and regions of consolidation or embolism (shown in blue) during the last 1 second of blood flow. (c) Shear rate history over the last 3 seconds prior to adhesion for platelets adhering in very high shear rate (γ_local_ >10000 s^-1^), high shear rate (γ_local_ ~ 4000 s^-1^) and moderate shear rate (γ_local_ <2000 s^-1^) regions.

Sample results demonstrating the potential of these methods are presented in Fig 6. The shear experienced by platelets when interacting with the thrombus surface is represented by the shear rate at the centre of the platelet 1 μm above the thrombus surface (Fig 6A). The shear rate 1 μm above the thrombus is comparatively lower than the shear rate measured directly on the surface due to the no-slip boundary condition. Taking the measurement at 1 μm also minimises the sensitivity of calculations to reconstruction of the thrombus surface. The shear rate contours show that several regions along the top of the thrombus experience peak shear rates in excess of 8000 s^-1^ and most regions across the thrombus surface have shear rates significantly greater than the wall shear rate (γ_wall_ = 1800 s^-1^). These elevated shear rates are likely to influence molecular pathways that control platelet activation and function. For example we recently described a metalloproteolytic loss of platelet glycoprotein VI, an adhesive receptor for collagen, upon exposure to transient, elevated shear stress [18].

The growth that the thrombus has experienced over the previous 1 second is plotted in Fig 6B. Regions of positive (red) growth correspond to regions of significant platelet adhesion. Regions of negative (blue) growth are regions where there has been loss due to either embolism or retraction of the thrombus. White regions indicate regions with no net change in thrombus size. As demonstrated in Fig 6B it is possible for multiple types of growth to occur on a given thrombus.

Three regions of interest corresponding to (1) very high shear rate (γ_local_ >10000 s^-1^), (2) moderately high shear rate (γ_local_ ~ 4000 s^-1^) and (3) moderate shear rate (γ_local_ <2000 s^-1^), as marked in Fig 6A & 6B, were identified on the thrombus surface and the shear time histories of these points investigated further. Fig 6C shows the shear rate experienced in the last 3 seconds prior to potential adhesion for these regions.

Platelets entering region (1) not only experienced higher shear rate (9783 s^-1^) near the thrombus surface, but immediately prior to potential adhesion experienced a very high temporal shear rate gradient. Examining region (1) in Fig 6B we see that this also corresponds to high growth or, in other words, these platelets tended to adhere. Platelets entering regions (2) and (3) were, at the surface, experiencing much lower shear rate and lower shear rate gradients but might have experienced higher shear rates as they passed over the thrombus earlier in the flow. The resultant growth for both these locations was low, indicating that platelets entering these regions at this time were unlikely to adhere.

A further 3 points were identified on the thrombus, based on the growth at these locations. Region (4) was a location of high growth while regions (5) and (6) were regions of thrombus loss, or negative growth. The shear rate profile in high growth region (4) is similar to that of region (1) as incoming platelets have experienced both high shear rates and high shear rate gradient. Both loss regions correspond to lower incoming shear rate. Overall from the current dataset, platelets which had experienced high shear gradients significantly contributed to regions of high growth. This indicates that the shear rate experienced by platelets is one of several factors that contributes to thrombus growth. The trends identified here are relatively simplistic as they are based on one thrombus at one timepoint; however they clearly demonstrate the capabilities of the developed techniques. Analysis of a much larger dataset is required to further elucidate the details of these mechanisms.

Currently the thrombus surface is being considered as a solid sealed surface with properties that do not significantly affect the shear rate experienced by platelets before they come in contact with the thrombus surface. Future work may also consider surface porosity as a function of time, the effect of active thrombin generation, fibrin generation and fibrinolysis, and the changes in surface shear rate in combination with platelet molecular markers. In addition to this, the role of RBCs on thrombus growth and the impact of platelets of varying size and shape will also be considered.

## Conclusions

A novel combination of *ex vivo* and *in silico* experiments have been presented to examine the role of flow mechanics in thrombus formation and individual platelet adhesion. Techniques have been developed which allow direct correlation of thrombus growth with the shear rate, and other flow properties, experienced by platelets prior to interaction with the growing thrombus surface. Within these techniques we developed a new method to extract instantaneous time-corrected reconstructions of a growing geometry from z-stacks when the geometry changes significantly during the z-stack acquisition time. Initial analysis using the techniques presented indicates a role for the temporal shear rate profile experienced by potentially adherent platelets in governing subsequent thrombus growth. We anticipate that these tools will enable molecular analysis of thrombus formation at an unprecedented level of resolution. Furthermore these tools will provide molecular insights that establish new avenues for development and assessment of novel therapeutic agents to improve standard antiplatelet therapies and lower bleeding risk.

## Supporting Information

S1 Text Extended Techniques. An extended description of some of the techniques used.
(DOCX)

S1 Table Shear Pathline Data. The shear data used to generate the individual pathlines.
(XLSX)
