# Supplementary material for: Methods to Determine the Lagrangian Shear Experienced by Platelets during Thrombus Growth
Source: PLoS One. 2015 Dec 14;10(12):e0144860. doi: 10.1371/journal.pone.0144860 (PMC4682839; doi:10.1371/journal.pone.0144860)
Supplement: S1 Text — An extended description of some of the techniques used. (DOCX) [file pone.0144860.s001.docx]

**Extended Techniques Supporting Information**

*Slice Thickness Selection*

The slice thickness was evaluated by acquiring z-stacks using 0.1 µm slices (pixel density of 0.31 µm/pixel). These slices were then used to produce surface reconstructions which utilised all the acquired slices, as well as every third and fifth slice to produce surfaces reconstructed from 0.1 µm, 0.3 µm and 0.5 µm slice thicknesses, respectively. Numerical simulations (discussed under flow techniques) were conducted to determine the differences in shear along the surface using the three different sectioning heights. By comparing the various shear fields, a slice thickness of 0.5 µm was selected due to the marginal difference in calculated shear and much faster acquisition times. Slice thickness greater than 0.5 µm did not adequately resolve platelet features.

*Surface Filtering*

The presence of “ripple” artefacts is independent of the number of slices used. To test and demonstrate these artefacts, a 10-mm high trapezoidal cone was mathematically generated and sliced into 1 mm thick slices (Figure 7). The corresponding slices had a binary threshold applied to determine the boundary of the trapezoidal cone at any given height and was reconstructed using Avizo. The resulting surface shows major ripple artefacts at frequencies that are a function of the slice thickness as the Avizo surface generation algorithm attempts to fit a surface which encloses all of the points from the given slices. To address this problem, in-house software was developed which utilises a Hamming window to filter the reconstructed surface from Avizo, producing a correlated surface. Filtering causes rippled edges to be replaced with straight edges as would be expected in a mathematically derived trapezoidal cone.


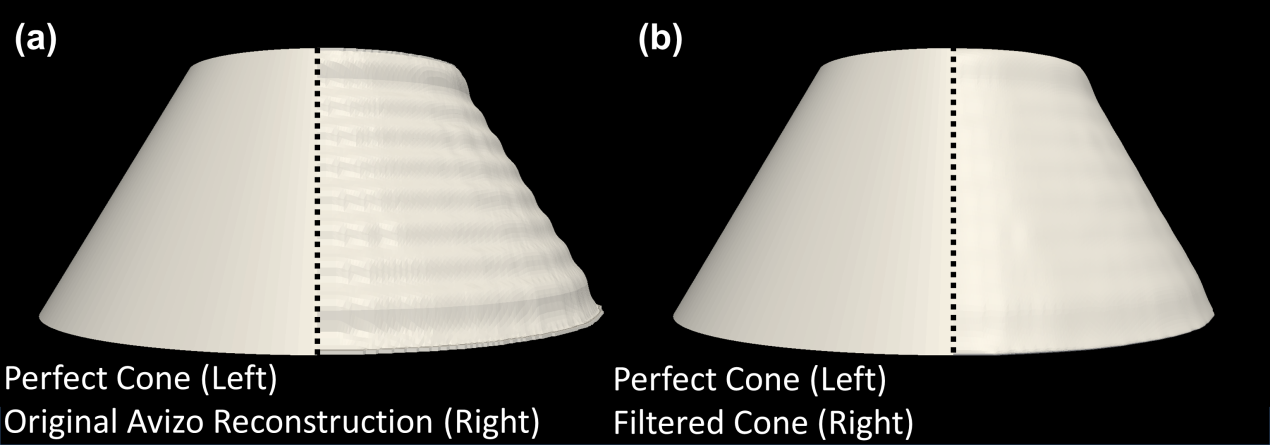


Figure 7. Comparison of a perfect cone, cone with artefacts and a filtered cone.

Techniques are demonstrated using a cone where the left side of the cone is the mathematically generated surface and the right side the reconstructed surface. (a) A mathematically derived trapezoidal cone surface (left) sliced at 1mm intervals is reconstructed as through Avizo’s reconstruction algorithm showing ripple artefacts (right). (b) Filtered reconstruction (right) produced by in-house developed filtering software (also at 1mm intervals) corrects for the ripple artefacts observed in a).

*Evaluation Of Current Morphing Techniques*

Several other existing morphing algorithms were evaluated ‎[1]‎[2]. The major limitation of these algorithms is the implementation of a mesh deformation transformation to achieve the transition between two objects. The objects in most cases must be similar in terms of features, which does not allow for embolism occurring between z-stacks. The other limitation is due to the colour correction of pixels. For example when transforming an individual white pixel to a black pixel the intermediate transformation frames contain the greyscale range between white and black. Normally when transitioning between two different images these particular limitations would not be an issue, however when dealing with images that contain very irregular objects that change rapidly over time these methods are no longer appropriate.

*Flow Experiments*

A polyhedral mesh consisting of (x, y, z) 500 x 250 x 50 rectangular elements was used in the numerical simulations. The flow was predominately in the x direction and required greater grid resolution in this direction. A grid resolution study was conducted, gradually increasing the number of mesh elements to 20 million. In comparing the highest mesh resolution (20 million elements) to that used during simulations (17 million elements), a difference of 0.1% in average velocity magnitude was observed while a difference of 1.03 x 10^-10^ in convergence levels occurred. However the minor gain in accuracy could not justify the increase in computational simulation time from 271 hours to more than 450 hours.

1. Steyvers M, Morphing techniques for manipulating face images, Behavior Research Methods, Instruments, & Computers. 1999; 31(2): 359-369.
2. Lee S, Wolberg G, Kyung-Yong C, Shin SY, Image metamorphosis with scattered feature constraints. Vis & Comp Graph. IEEE. 1996; 2(4): 337-354.
